# Supplementary material for: HIVprotI: an integrated web based platform for prediction and design of HIV proteins inhibitors
Source: J Cheminform. 2018 Mar 9;10:12. doi: 10.1186/s13321-018-0266-y (PMC5845081; doi:10.1186/s13321-018-0266-y)
Supplement: Supplementary file 2 — Additional file 2. Source code of HIVProtI web server. [file 13321_2018_266_MOESM2_ESM.zip › HIVprotI_Source-code/hivprotI/weka-3-6-6/doc/weka/core/package-summary.html]

weka.core


---


|  |  |  |
| --- | --- | --- |
| **Overview** \| **Package** \| Class \| **Tree** \| **Deprecated** \| **Index** \| **Help** \| **Weka's home** | |  |
| **PREV PACKAGE**   **NEXT PACKAGE** | **FRAMES**    **NO FRAMES**     **All Classes** |  |


---

## Package weka.core

| **Interface Summary** | |
| --- | --- |
| **AdditionalMeasureProducer** | Interface to something that can produce measures other than those calculated by evaluation modules. |
| **CapabilitiesHandler** | Classes implementing this interface return their capabilities in regards to datasets. |
| **Copyable** | Interface implemented by classes that can produce "shallow" copies of their objects. |
| **DistanceFunction** | Interface for any class that can compute and return distances between two instances. |
| **Drawable** | Interface to something that can be drawn as a graph. |
| **EnvironmentHandler** | Interface for something that can utilize environment variables. |
| **JythonObject** | An indicator interface for Jython objects. |
| **JythonSerializableObject** | An indicator interface for serializable Jython objects. |
| **Matchable** | Interface to something that can be matched with tree matching algorithms. |
| **MultiInstanceCapabilitiesHandler** | Multi-Instance classifiers can specify an additional Capabilities object for the data in the relational attribute, since the format of multi-instance data is fixed to "bag/NOMINAL,data/RELATIONAL,class". |
| **OptionHandler** | Interface to something that understands options. |
| **Randomizable** | Interface to something that has random behaviour that is able to be seeded with an integer. |
| **RevisionHandler** | For classes that should return their source control revision. |
| **Summarizable** | Interface to something that provides a short textual summary (as opposed to toString() which is usually a fairly complete description) of itself. |
| **TechnicalInformationHandler** | For classes that are based on some kind of publications. |
| **Undoable** | Interface implemented by classes that support undo. |
| **WeightedInstancesHandler** | Interface to something that makes use of the information provided by instance weights. |

| **Class Summary** | |
| --- | --- |
| **AbstractStringDistanceFunction** | Represents the abstract ancestor for string-based distance functions, like EditDistance. |
| **AlgVector** | Class for performing operations on an algebraic vector of floating-point values. |
| **AllJavadoc** | Applies all known Javadoc-derived classes to a source file. |
| **Attribute** | Class for handling an attribute. |
| **AttributeExpression** | A general purpose class for parsing mathematical expressions involving attribute values. |
| **AttributeLocator** | This class locates and records the indices of a certain type of attributes, recursively in case of Relational attributes. |
| **AttributeStats** | A Utility class that contains summary information on an the values that appear in a dataset for a particular attribute. |
| **BinarySparseInstance** | Class for storing a binary-data-only instance as a sparse vector. |
| **Capabilities** | A class that describes the capabilites (e.g., handling certain types of attributes, missing values, types of classes, etc.) of a specific classifier. |
| **ChebyshevDistance** | Implements the Chebyshev distance. |
| **Check** | Abstract general class for testing in Weka. |
| **CheckGOE** | Simple command line checking of classes that are editable in the GOE. Usage:  `CheckGOE -W classname -- test options`   Valid options are: |
| **CheckOptionHandler** | Simple command line checking of classes that implement OptionHandler. Usage:  `CheckOptionHandler -W optionHandlerClassName -- test options`   Valid options are: |
| **CheckScheme** | Abstract general class for testing schemes in Weka. |
| **CheckScheme.PostProcessor** | a class for postprocessing the test-data |
| **ClassDiscovery** | This class is used for discovering classes that implement a certain interface or a derived from a certain class. |
| **ClassDiscovery.StringCompare** | compares two strings. |
| **ClassloaderUtil** | Utility class that can add jar files to the classpath dynamically. |
| **ContingencyTables** | Class implementing some statistical routines for contingency tables. |
| **Copyright** | A class for providing centralized Copyright information. |
| **Debug** | A helper class for debug output, logging, clocking, etc. |
| **Debug.Clock** | A little helper class for clocking and outputting times. |
| **Debug.DBO** | contains debug methods |
| **Debug.Log** | A helper class for logging stuff. |
| **Debug.Random** | This extended Random class enables one to print the generated random numbers etc., before they are returned. |
| **Debug.SimpleLog** | A little, simple helper class for logging stuff. |
| **Debug.Timestamp** | A class that can be used for timestamps in files, The toString() method simply returns the associated Date object in a timestamp format. |
| **EditDistance** | Computes the Levenshtein edit distance between two strings. |
| **Environment** | This class encapsulates a map of all environment and java system properties. |
| **EuclideanDistance** | Implementing Euclidean distance (or similarity) function.    One object defines not one distance but the data model in which the distances between objects of that data model can be computed.    Attention: For efficiency reasons the use of consistency checks (like are the data models of the two instances exactly the same), is low.    For more information, see:    Wikipedia. |
| **FastVector** | Implements a fast vector class without synchronized methods. |
| **FindWithCapabilities** | Locates all classes with certain capabilities. |
| **GlobalInfoJavadoc** | Generates Javadoc comments from the class's globalInfo method. |
| **Instance** | Class for handling an instance. |
| **InstanceComparator** | A comparator for the Instance class. |
| **Instances** | Class for handling an ordered set of weighted instances. |
| **Javadoc** | Abstract superclass for classes that generate Javadoc comments and replace the content between certain comment tags. |
| **Jython** | A helper class for Jython. |
| **ListOptions** | Lists the options of an OptionHandler |
| **ManhattanDistance** | Implements the Manhattan distance (or Taxicab geometry). |
| **MathematicalExpression** | Class for evaluating a string adhering the following grammar: |
| **Matrix** | **Deprecated.** *Use `weka.core.matrix.Matrix` instead - only for backwards compatibility.* |
| **Memory** | A little helper class for Memory management. |
| **NormalizableDistance** | Represents the abstract ancestor for normalizable distance functions, like Euclidean or Manhattan distance. |
| **Optimization** | Implementation of Active-sets method with BFGS update to solve optimization problem with only bounds constraints in multi-dimensions. |
| **Option** | Class to store information about an option. |
| **OptionHandlerJavadoc** | Generates Javadoc comments from the OptionHandler's options. |
| **PropertyPath** | A helper class for accessing properties in nested objects, e.g., accessing the "getRidge" method of a LinearRegression classifier part of MultipleClassifierCombiner, e.g., Vote. |
| **PropertyPath.Path** | Contains a (property) path structure |
| **PropertyPath.PathElement** | Represents a single element of a property path |
| **ProtectedProperties** | Simple class that extends the Properties class so that the properties are unable to be modified. |
| **Queue** | Class representing a FIFO queue. |
| **RandomVariates** | Class implementing some simple random variates generator. |
| **Range** | Class representing a range of cardinal numbers. |
| **RelationalLocator** | This class locates and records the indices of relational attributes, |
| **RevisionUtils** | Contains utility functions for handling revisions. |
| **SelectedTag** | Represents a selected value from a finite set of values, where each value is a Tag (i.e. |
| **SerializationHelper** | A helper class for determining serialVersionUIDs and checking whether classes contain one and/or need one. |
| **SerializedObject** | Class for storing an object in serialized form in memory. |
| **SingleIndex** | Class representing a single cardinal number. |
| **SparseInstance** | Class for storing an instance as a sparse vector. |
| **SpecialFunctions** | Class implementing some mathematical functions. |
| **Statistics** | Class implementing some distributions, tests, etc. |
| **Stopwords** | Class that can test whether a given string is a stop word. |
| **StringLocator** | This class locates and records the indices of String attributes, recursively in case of Relational attributes. |
| **SystemInfo** | This class prints some information about the system setup, like Java version, JVM settings etc. |
| **Tag** | A `Tag` simply associates a numeric ID with a String description. |
| **TechnicalInformation** | Used for paper references in the Javadoc and for BibTex generation. |
| **TechnicalInformationHandlerJavadoc** | Generates Javadoc comments from the TechnicalInformationHandler's data. |
| **Tee** | This class pipelines print/println's to several PrintStreams. |
| **TestInstances** | Generates artificial datasets for testing. |
| **Trie** | A class representing a Trie data structure for strings. |
| **Trie.TrieIterator** | Represents an iterator over a trie |
| **Trie.TrieNode** | Represents a node in the trie. |
| **Utils** | Class implementing some simple utility methods. |
| **Version** | This class contains the version number of the current WEKA release and some methods for comparing another version string. |

| **Enum Summary** | |
| --- | --- |
| **Capabilities.Capability** | enumeration of all capabilities |
| **RevisionUtils.Type** | Enumeration of source control types. |
| **TechnicalInformation.Field** | the possible fields |
| **TechnicalInformation.Type** | the different types of information |

| **Exception Summary** | |
| --- | --- |
| **NoSupportForMissingValuesException** | Exception that is raised by an object that is unable to process data with missing values. |
| **UnassignedClassException** | Exception that is raised when trying to use some data that has no class assigned to it, but a class is needed to perform the operation. |
| **UnassignedDatasetException** | Exception that is raised when trying to use something that has no reference to a dataset, when one is required. |
| **UnsupportedAttributeTypeException** | Exception that is raised by an object that is unable to process some of the attribute types it has been passed. |
| **UnsupportedClassTypeException** | Exception that is raised by an object that is unable to process the class type of the data it has been passed. |
| **WekaException** | Class for Weka-specific exceptions. |

---


|  |  |  |
| --- | --- | --- |
| **Overview** \| **Package** \| Class \| **Tree** \| **Deprecated** \| **Index** \| **Help** \| **Weka's home** | |  |
| **PREV PACKAGE**   **NEXT PACKAGE** | **FRAMES**    **NO FRAMES**     **All Classes** |  |


---
